# Global potential distribution of *Drosophila suzukii* (Diptera, Drosophilidae)

**DOI:** 10.1371/journal.pone.0174318

**Published:** 2017-03-21

**Authors:** Luana A. dos Santos, Mayara F. Mendes, Alexandra P. Krüger, Monica L. Blauth, Marco S. Gottschalk, Flávio R. M. Garcia

**Affiliations:** 1 Pós-Graduação em Entomologia, Instituto de Biologia, Universidade Federal de Pelotas, Pelotas, Rio Grande do Sul, Brazil; 2 Pós-Graduação em Biologia Animal, Instituto de Biologia, Universidade Federal de Pelotas, Pelotas, Rio Grande do Sul, Brazil; 3 Pós-Graduação em Fitossanidade, Faculdade de Agronomia Eliseu Maciel, Universidade Federal de Pelotas, Pelotas, Rio Grande do Sul, Brazil; 4 Departamento de Ecologia, Zoologia e Genética, Instituto de Biologia, Universidade Federal de Pelotas, Pelotas, Rio Grande do Sul, Brazil; CNRS, FRANCE

## Abstract

*Drosophila suzukii* (Matsumura) is a species native to Western Asia that is able to pierce intact fruit during egg laying, causing it to be considered a fruit crop pest in many countries. *Drosophila suzukii* have a rapid expansion worldwide; occurrences were recorded in North America and Europe in 2008, and South America in 2013. Due to this rapid expansion, we modeled the potential distribution of this species using the *Maximum Entropy Modeling* (MaxEnt) algorithm and the *Genetic Algorithm for Ruleset Production* (GARP) using 407 sites with known occurrences worldwide and 11 predictor variables. After 1000 replicates, the value of the average area under the curve (AUC) of the model predictions with 1000 replicates was 0.97 for MaxEnt and 0.87 for GARP, indicating that both models had optimal performances. The environmental variables that most influenced the prediction of the MaxEnt model were the annual mean temperature, the maximum temperature of the warmest month, the mean temperature of the coldest quarter and the annual precipitation. The models indicated high environmental suitability, mainly in temperate and subtropical areas in the continents of Asia, Europe and North and South America, where the species has already been recorded. The potential for further invasions of the African and Australian continents is predicted due to the environmental suitability of these areas for this species.

## Introduction

Drosophilidae (Diptera) consists of approximately 4,200 species, but few are considered pests because they preferentially breed in decaying plant material. *Drosophila suzukii* (Matsumura) (Diptera: Drosophilidae), the spotted wing drosophila (SWD), is a species characterized as a fruit pest due to its unique ability to pierce soft-skinned fruits during egg laying, a polyphagous habit and a preference for fresh fruits [1]. The first recorded occurrence outside Asia was in 1980, in Oahu, Hawaii (USA). *Drosophila suzukii* was subsequently found on other Hawaiian Islands [2]. Since then, the geographical distribution of *D*. *suzukii* has expanded rapidly.

In 2008, *D*. *suzukii* reached the North American continent, where it was reported in California, USA [3]. Its distribution in USA has since grown considerably, reaching the Pacific Division and the states of Idaho, Montana and Utah in the Mountain Division of West Region, and the states of Northeast and the South Regions, except Oklahoma and Texas [4]. In Canada, *D*. *suzukii* has been recorded in the provinces of British Columbia, Alberta, Manitoba, Ontario, Quebec, New Brunswick, Nova Scotia, Prince Edward Island and Newfoundland [5]. In 2011, the *North American Plant Protection Organization* (NAPPO) recorded this species in Mexico [6].

In Europe, *D*. *suzukii* was also detected in Spain and Italy in 2008; and soon thereafter, it was reported in France, Austria, Germany, Belgium, Croatia, Slovenia, Ireland, the United Kingdom and Switzerland. In 2012, the species was recorded in the westernmost part of the Iberian Peninsula and in Portugal. The *European Plant Protection Organization* (EPPO) has estimated that this species is now present in all European countries [7].

The first record of this species in South America occurred in 2013, in the South of Brazil [8]. In 2014, *D*. *suzukii* was recorded in the southeastern and central regions of the country [9, 10], approximately 1700 km from its first recorded location in the country.

In view of the broad and rapidly expanding distribution of *D*. *suzukii* on most continents, this study aimed to determine its potential worldwide geographic distribution using distribution models derived from the existing occurrence data of the species. The generated models will allow us to predict areas at potential risk for the establishment of this species, based on abiotic conditions [11].

## Material and methods

### Obtaining records of the occurrence of *D*. *suzukii*

Sites with known occurrence were first obtained from the TaxoDros database v.1.04 (dated 2015/03) [12] up to September 2015. An additional literature review was performed to find studies not available in TaxoDros, such as, the records from the Neotropical Region. The reported occurrence sites and geographic coordinates were checked and validated by consulting the original literature sources and entering the geographic coordinates in Google Maps (www.google.com/maps), thereby avoiding dubious geographic coordinates and uncertain locations. The references used to create the database are listed in the Supporting Information (S1 File).

All coordinates were double-checked to ensure their accuracy in the raster of environmental variables used in the analysis using the QGIS 2.10.1 application (http://www.qgis.org/pt_BR/site).

### Obtaining and selecting environmental variables used in the geographical distribution models

The environmental variables used for computer modeling were the Bioclim variables and altitude measurements obtained from the WorldClim Global Climate Database 1.3 (http://www.worldclim.org/) [13].

To avoid using autocorrelated climatic variables, the variables were standardized, and then, a Principal Component Analysis (PCA) was performed with the 19 variables [14] using the Past 2.17c application [15]. Variables associated with the main components that had eigenvalues greater than 1 were selected. Their associations were checked using a Spearman correlation analyses and those where rs > |0.75| were selected (Supporting Information–S1 and S2 Tables). Finally, ten bioclimate environmental variables were selected: annual mean temperature (Bio-1), mean diurnal range (Bio-2), temperature seasonality (Bio-4), maximum temperature of the warmest month (Bio-5), minimum temperature of the coldest month (Bio-6), annual range of temperature (Bio-7), mean temperature of the coldest quarter (Bio-11), annual precipitation (Bio-12), precipitation of the driest quarter (Bio-17) and precipitation of the warmest quarter (Bio-18). In addition, the variable altitude (Alt) was also included (note that Alt was not analyzed for multicollinearity but was included because it is not a climatic variable).

To assess whether the values of the environmental variables in the sites where *D*. *suzukii* already occurs were spatially autocorrelated, we calculated the value of Moran’s I [16] for each of the eleven environmental variables previously described using the package Ade4 v.1.7–4 [17] from the R 3.1.3 release (https://cran.r-project.org/). The test significance was obtained by a Monte Carlo test with 1000 randomizations. No spatial autocorrelation was found for any of the selected variables (Supporting Information–S3 Table).

### Algorithms used and the performance evaluation of the models obtained

We used two algorithms: *Maximum Entropy Modeling* (MaxEnt) and the *Genetic Algorithm for Ruleset Production* (GARP), both of which require only occurrence data [18]. To conduct the computer modeling, we used a resolution of 5 arc minutes. The predictive performance of the generated models was evaluated by reconstructing 1000 bootstrap replicas. In each replica, 50% of the records randomly selected by the two algorithms were retained as test data. To evaluate each generated model, the *Receiver Operating Characteristic* (ROC) curves were constructed and the *Area Under the Curve* (AUC) was calculated. The AUC value ranges from 0 to 1, where 1 indicates a perfect ability to discriminate between the omission of areas with records and the overlap of occupied areas.

Furthermore, the AUC also serves as a measure for evaluating the independent model at a chosen cut-off threshold [19, 20, 21]. To development the models, we used the software Maximum Entropy Modeling v.3.3.3k (MaxEnt) (available at http://www.cs.princeton.edu/~schapire/maxent/) and openModeller 1.5.0 (available at http://openmodeller.sourceforge.net/).

### Thresholds for defining areas with predicted presence of *D*. *suzukii*

For the MaxEnt algorithm, the threshold for defining areas with the presence or absence of *D*. *suzukii* was the average of the values of the *Minimum Training Presence* (*MTP*) of the 1000 generated models. For the GARP algorithm, the threshold adopted was a 70% probability of presence of the species, as suggested in openModeller.

## Results

**Fig 1:**
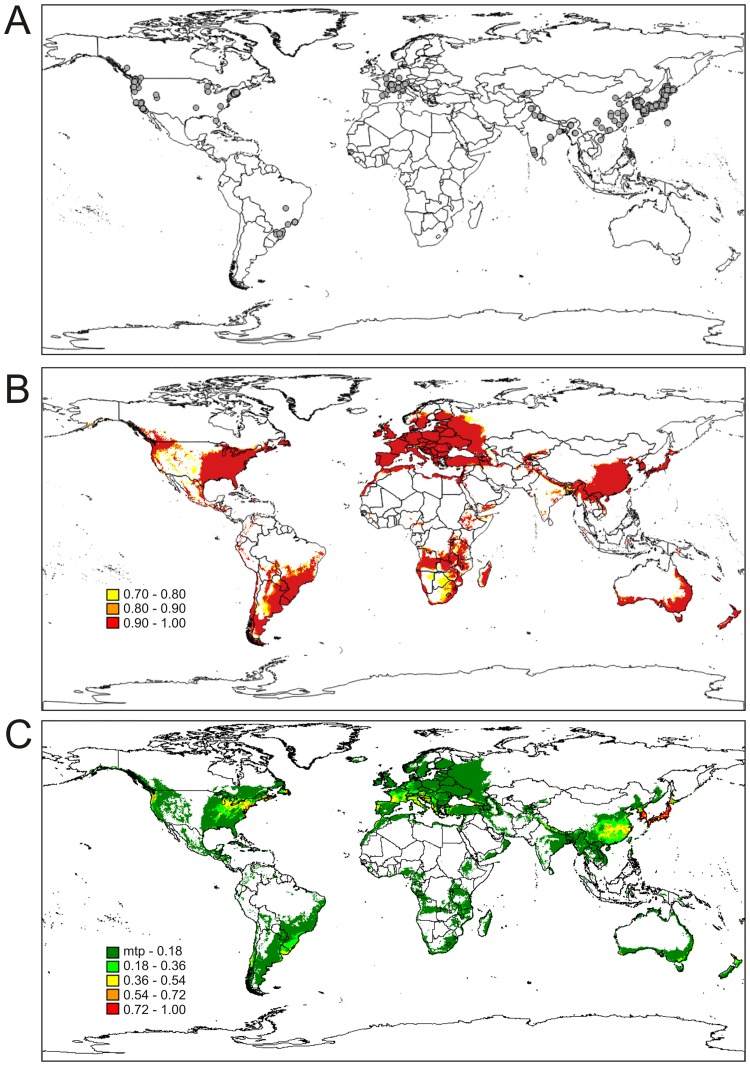
Potential distribution of *D*. *suzukii*. (A) Known existing sites of occurrence of *D*. *suzukii* used to generate the predictive models. (B) Predictive model of the geographic distribution of *D*. *suzukii* generated by the GARP algorithm. (C) Predictive model of the geographic distribution of *D*. *suzukii* generated by the MaxEnt algorithm. The legend indicates low (0) and high (1) environmental suitability for *D*. *suzukii*.

The database included a total of 407 occurrence sites of *D*. *suzukii* (Fig 1A).

This species is currently distributed in the Asian, European, and North and South American continents. The models showed optimal performance; the mean AUC value for the GARP algorithm was 0.89, while for MaxEnt it was 0.97.

The projections of the potential distribution of *D*. *suzukii* in both models under their respective thresholds were quite similar (Fig 1B and 1C), indicating high reliability in the generated predictions. However, the GARP model was somewhat more restrictive in predicting the potential area for establishment of *D*. *suzukii* except in South Africa, where its prediction of the potential distribution area was larger.

In Asia, the endemic area of the species, the countries that have a predicted wider potential distribution of *D*. *suzukii* are Japan, North and South Korea, eastern China, Vietnam, Cambodia, Laos, a narrow stretch of northern India, Pakistan, Nepal, part of Thailand, Myanmar, Bhutan, Bangladesh, Nepal, parts of Yemen and Oman, Tajikistan, eastern Uzbekistan, and countries near the Mediterranean such as Turkey, Georgia, northwest Iran, Azerbaijan and the Mediterranean coasts of Lebanon and Syria. Of these, the regions with the highest suitability in the model developed with the MaxEnt algorithm are the countries in East Asia, including Japan, North and South Korea and the entire coast of China, in addition to Georgia, which lies on the border between Europe and Asia.

Almost all of Europe was identified as an area of potential suitability of this species, except for northern Sweden, Finland, and northwest Russia. The countries with higher suitability in the models developed using MaxEnt and GARP were Portugal, France, Italy, Austria, Budapest, Greece, Albania, Switzerland, southern Germany and extreme northern Spain. Between Asia and Europe there is a north-south range where the models did not predict the presence of the species, delimiting a discontinuity in the potential distribution of *D*. *suzukii*.

In North America, the areas for the potential suitability of *D*. *suzukii* lie in a wide range along most of the Atlantic coastline and in a narrow range on the Pacific coast, widening toward the northwestern USA and southwestern Canada.

In South America, the potential areas for the distribution of *D*. *suzukii* as predicted by the MaxEnt algorithm extend the entire length of the Atlantic coast, which differed from the GARP algorithm. Nevertheless, the models generated by both algorithms indicate that the central region of southern Brazil, the southern half of Paraguay, all of Uruguay and the regions to the east and south of Argentina as potential distribution areas. On the Pacific coast, the entire coastline of Chile is indicated as a potential distribution area of the species. The areas of greatest environmental suitability for *D*. *suzukii* are in southern Chile, Uruguay, on the south coast and in southern Brazil, and along small range on the northern coast of Argentina.

To date, there are no records of *D*. *suzukii* in Africa or Oceania. However, our models indicate that high environmental suitability for this species exists in some regions of Africa such as eastern and northwestern Madagascar, the southern region and the Lakes region in eastern South Africa, southwest Namibia, a central range of Angola, the coastal region of the Democratic Republic of the Congo, northern and southern Gabon, the coastal and central regions of Cameroon and Nigeria, western Ethiopia, southern South Sudan, Uganda, southern and northern Tanzania, northern Mozambique, Zambia, central Zimbabwe, the Mediterranean coastal region and the Atlantic Western Sahara, Morocco, northern Algeria, coastal Tunisia, Libya and Egypt. The area of greatest environmental suitability indicated by the model developed with the MaxEnt algorithm was the coast of Morocco.

A narrow band of Oceania was identified as suitable for the establishment of *D*. *suzukii* in southern, northern and eastern Australia. New Zealand was also indicated as environmentally suitable for the establishment of this species. The regions of greater environmental suitability, indicated by the model developed with the MaxEnt algorithm, were the North Island of New Zealand, Tasmania, and a narrow range in the southeast and southwest of Australia. These areas may be more susceptible to future invasions by *D*. *suzukii*.

### Environmental variables determining the distribution of *D*. *suzukii*

**Fig 2:**
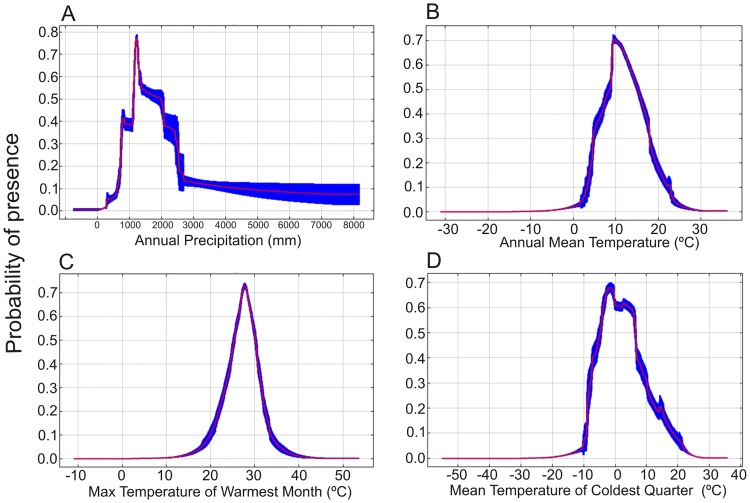
Average response curves of the main predictor variables of the distribution model of *D*. *suzukii* generated by the MaxEnt algorithm. (A) Annual precipitation (Bio-12), (B) annual mean temperature (Bio-1), (C) maximum temperature of the warmest month (Bio-5), and (D) mean temperature of the coldest quarter (Bio-11) used to estimate the probability of occurrence of *D*. *suzukii*. The red lines show the average of probability values from 1000 iterations using randomized input, and the blue lines show the standard deviations.

The environmental variables that contributed most to the construction of the potential distribution model of *D*. *suzukii* with the MaxEnt algorithm were annual precipitation (Bio-12) (27.1%), annual mean temperature (Bio-1) (contribution of 20.9%), maximum temperature of the warmest month (Bio-5) (9.7%) and mean temperature of the coldest quarter (Bio-11) (9.2%). The standard curves of the distribution model of *D*. *suzukii* (Fig 2) indicate that the probability of occurrence of this species decreases considerably in regions in which the mean temperature of the coldest quarter is below 10°C and the maximum temperature of the warmest month above 33°C. The probability of occurrence of *D*. *suzukii* increases in areas with an annual mean temperature between 5°C and 20°C. Regarding rainfall, the standard curves of the model indicate that the annual precipitation also strongly influences the distribution of the species, and it is more likely to occur in areas that experience annual rainfall of between 500 and 2,500 mm.

## Discussion

The aim of our study was to detect suitable areas of establishment of *D*. *suzukii* using inductive distribution modeling and the abiotic variables of the sites where the species was already recorded, assisting the studies with non-inductive approach, carried out with modeling based on physiological information of the species, using an inductive approach. As well discussed by Venette *et al*. [22], the inductive methods require little knowledge of the biology of the species and the result may suggest complex interactions between species and its environment. The authors also consider that the inductive approach use a statistical methodology to generate a forecast of niche distribution which not always have a biological support since population processes are not considered. We consider the inductive and non-inductive approaches as complementary and below we make some considerations that explain why we adopted the inductive modelling methodology.

An advantage of this approach was that the environmental conditions considered suitable for the species were estimated from the environmental parameters of the locations where the species has already been recorded rather than from the responses in simulated conditions in a laboratory, where the environmental variables tend to be constant throughout the assays. In addition, laboratory assays are usually conducted with one or only a few different fly strains, which can reduce the range of responses from the different populations. However, this advantage may not hold when record occurrences are ephemeral, such as the response to a very specific temporal condition, or stochastic, or when no population is established at a recorded site. An example for *Drosophila* was of the invasive species *D*. *malerkotliana* Parshad and Paika, which was once recorded in Porto Alegre, South Brazil [23]; however, since then, no other record of the species has been made in this location. Because the geo-referencing of some records may not be reliable, the MaxEnt and GARP algorithms excluded 5% of the sites with outlying environmental conditions from the train dataset to increase the model confidence [24].

The use of abiotic variables to generate the distribution modeling was supported by the influence of these factors on the population sizes of some Drosophilidae species [25, 26]. Biological variables such as host plants and competing or predator species, despite their importance for species establishment, need further study for *D*. *suzukii* worldwide. Furthermore, regarding the use of the plant hosts, *D*. *suzukii* has been shown to be polyphagous and is not restricted to thin-skinned fruit [27, 28], which hinders the use of host plants as a variable for modeling.

Recently, based on life-cycle stages of *D*. *suzukii* [29], Benito *et al*. [30] used temperatures responses established from physiological assays to create a model of distribution with Climex. Their results showed a potential distribution pattern in Brazil that was quite similar to our predictions but became more restricted when cold and heat stress temperatures where used as thresholds (stress temperatures were obtained in laboratory assays suggesting the upper and lower limits to the populations survival [29]). For example, their model excluded the northeast Atlantic coast, where the species has not yet been recorded. When we compared our MaxEnt/GARP global modeling with the global modeling of Benito *et al*. [30], we observed that their predictions were more restrictive and did not include some areas where the species is already established, such as India and the Pacific coast of the USA. Therefore, the distribution models generated with temperature thresholds based on developmental stages did not agree with the actual distribution of the species. In turn, Gutierrez *et al*. [31] has generated models of suitability for *D*. *suzukii* in USA, Mexico, Europe and northern Africa, using physiological data obtained from different studies and the Physiologically-Based Demographic Model (PBDM) method. The authors has suggested the establishment of the species, where the most suitable areas in the eastern EUA were the southeast of the country and the coast of the Gulf of Mexico; in Mexico, the south, the coast of the Gulf of Mexico and of the Pacific coast of the country; In Europa and north Africa were the Mediterranean coast of Europe and the Atlantic coast of Portugal, Spain, France and the Mediterranean coast of Africa. Our models, especially MaxEnt, have suggested that the distribution of the species covers the eastern EUA, as suggested by Gutierrez et al. [31]. However, the most suitable area, according our models, has been the northeast (Michigan, Indiana, Ohio, Pensilvania, Delaware, New York, Rhode Island, Maine, West Virginia and Virginia) and the northwest of the country (California, Oregon and Washington). As well, our models have not indicated high suitable area at Mexico, but Gutierrez et al. [31] did. At Europe and northern of Africa, our models had greater similarities to that generated by Gutierrez et al. [31], however we have suggested higher suitability in Portugal, southern France, center and northern Italy, besides the coast of Adriatic Sea. The western and eastern coast of the Black Sea have had suitable area in both of our models and from Gutierrez *et al*. [31]. Although our models have shown that temperate and subtropical regions have potential areas of distribution of the species, the temperate region was more suitable, differing from what proposed by Gutierrez et al. [31] which highlighted the subtropical region as the most suitable for the species.

Some physiological parameters of the *D*. *suzukii* have been used to predict their distribution by considering not only high environmental suitability but also to establish conditions for managing the species [32]. In this sense, the MaxEnt algorithm can be used to suggest important environmental variables to *D*. *suzukii*. In our analysis, the annual precipitation variable had the most influence on the model. Variables associated with hydric stress were not considered in studies of Benites *et al*. [30], Wiman *et al*. [32] and Asplen *et al*. [33], but Tochen *et al*. [34] verified that in occasions with low relative humidity the sampling of *D*. *suzukii* decrease and Gutierrez *et al*. [31] observed that low relative humidity may be limiting in arid regions. The studies that included physiological traits properly approach the effects of temperature, corroborating the variables that the MaxEnt algorithm indicated as having a high influence in the distribution of *D*. *suzukii*: the annual mean temperature, maximum temperature of the warmest month and mean temperature of the coldest quarter. The maximum temperature of the warmest month (values above approximately 33°C) in the areas with higher environmental suitability estimated by the MaxEnt model is similar to the temperature observed as the heat stress threshold for *D*. *suzukii* as reported by Asplen *et al*. [33] (which varied from 29 to 33°C, depending on the biological parameters considered). However, our estimates of the mean temperature of the coldest quarter (lower values of approximately -10°C) are inferior to the temperatures reported by the same authors (which varied from 10 to -2°C). This wide difference can be justified by the ability of the species to enter diapause [35] in regions with temperatures below the reported cold stress temperatures.

Under these circumstances, the potential distribution of *D*. *suzukii* predicted by both generated models mainly comprises regions of subtropical or warm temperate climates with high rainfall throughout the whole year or during part of the year. These regions are identified as having a Cf (temperate mesothermal climates without dry seasons) or Df (cold continental climates without dry seasons) climates in the Köppen-Geiger classification—particularly Dfa (hot-summer humid continental climate) and/or Dfb (warm-summer humid continental climate). On the Asian continent, the models indicate that regions with humid subtropical climates have a greater probability of *D*. *suzukii* occurrence. These areas are characterized by dry and cold winters alternating with wet summers, where the mean monthly maximum temperature is 20°C and the mean monthly minimum temperature is 0°C [36]. On the Indian subcontinent, the models indicate areas of environmental suitability in regions with a humid subtropical climate in northern India: mountainous areas with extremely humid conditions due to heavy rains [37]. There are also areas predicted for the species in northeast Pakistan, despite the fact that seasons there alternate between periods of drought and heavy rains [38].

Currently, *D*. *suzukii* is widely distributed on the European continent [7], which has a temperated prevailing climate with well-defined seasons. The areas of greatest environmental suitability are in the west and south, which are dominated by a temperate Mediterranean climate, with hot, dry summers and wet, unstable winters. Our models support the hypothesis that *D*. *suzukii* is distributed across the continent [7].

*Drosophila suzukii* has a high thermal tolerance and a high potential for colonizing different habitats; it is able to withstand both hot summers in Spain and cold areas, such as mountainous regions in Japan and the Alps [7]. Its occurrence was confirmed in mountainous regions by Tonina *et al*. [39], and its survival in regions with mean daily temperatures below 11°C shows both its adaptive capacity to cold conditions and its tolerance of wide temperature range typical of mountains.

In North America, again, the areas with greater potential *D*. *suzukii* distribution are classified as having Cf and Df climates. These areas extend across down the entire Atlantic coast and along a narrow range in western Canada. Areas that are inadequate or only marginally suitable for the species are those with a semi-arid climate (BSh and BSk, respectively hot semi-arid climate and cold semi-arid climate), where humidity and rainfall are low.

The developed distribution models indicate areas with high environmental suitability in western Mexico, where the presence of *D*. *suzukii* was recently confirmed by Lasa and Tadeo [40]. These areas have a humid climate, with average annual temperatures of 20°C, warm winters and rainfall evenly distributed throughout the year.

In recent years, the presence of *D*. *suzukii* in South America has become more constant, primarily due to the climatic conditions of Brazil, where a humid subtropical climate predominates, with humid summers, cold winters and abundant rainfall distributed throughout the year [41]. The equatorial and tropical areas of South America do not seem to sustain populations of *D*. *suzukii*. Nonetheless, the models indicate potential distribution areas across the eastern extension of the Atlantic coast, including in some northeastern regions of Brazil, despite the semi-arid climate. Some states, such as Rio Grande do Norte, have average annual temperatures above 26.5°C and receive less than 600 mm rainfall annually. In other words, hot, dry regions with scarce rainfall are less suitable for the establishment of the species [41]. As noted by the models, potential distribution areas for *D*. *suzukii* extend from central to southern Brazil, confirming the results found by Paula *et al*. [10] in the Cerrado region of Brazil, and by Bitner-Mathé *et al*. [9] and Geisler *et al*. [27] in southern Brazil, where the average temperature varies between 14.8°C and 23.0°C. We also found greater environmental suitability in the models in regions with areas of subtropical Atlantic forest, as was recorded by Deprá *et al*. [8] for the states of Santa Catarina and Rio Grande do Sul in southern Brazil.

Moreover, there were areas of potential distribution and higher environmental suitability for *D*. *suzukii* in eastern and southern Argentina, in accordance with the record of the species in Argentina by Sandatino *et al*. [42]. We also found areas suitable for the species throughout Uruguay, which is dominated by a subtropical climate with well-defined seasons and an extremely wide annual temperature range: from negative values in the winter to 40°C in the summer. *Drosophila suzukii* was already been record in southern Uruguay, in the region of Montevideo by González *et al*. [43]. In addition, our models predict potential distribution areas in the southern half of Paraguay and on the Pacific coast, along the coastline of Chile. The most suitable areas for the species are in southern Chile, where Vilela and Mori [44] suggested its presence, which was confirmed by Medina-Muñoz *et al*. [45].

The models also show some areas of environmental suitability for *D*. *suzukii* in Costa Rica and Ecuador, supporting Hauser [3], who reports the record of the species before 2000 through some personal communications to him. However, to date, there are no voucher specimens of the species collected in these countries.

In general, *D*. *suzukii* occupies areas with a wide temperature range and exhibits a gradual process of acclimation, which is an important adaptive characteristic [46]. According to Calabria *et al*. [47], the optimum temperature for *D*. *suzukii* development is between 20°C and 25°C, and temperatures above 30°C cause a reduction in longevity. Kimura [48] reported that temperatures between 32.2°C and 32.7°C have a negative effect on adults of *D*. *suzukii* and were lethal to 50% of the individuals studied. However, Tochen *et al*. [29] found that the optimal developmental temperature for the species was 28.1°C.

The adults of *D*. *suzukii* tolerate low temperatures. Our MaxEnt algorithm model suggests that it do not establish in areas with average annual temperatures is below 0°C and with mean temperature of coldest quarter below -10°C, in agreement to some physiological studies. According to Jakobs *et al*. [49], temperatures below 0°C decrease their reproductive activity and their survivability diminishes. Kimura [48] report that *D*. *suzukii* could survive at temperature above -5°C. In contrast, Dalton *et al*. [46] verified that *D*. *suzukii* is unlikely to survive at temperatures below 10°C. The different responses in experimental procedures may be related with the strain of *D*. *suzukii* studied, how the strain are reared in laboratory or even when or from where the strain were caught. For *D*. *suzukii*, two morphologies associated with summer and winter seasons have been studied, which responds differently to temperature. Shearer *et al*. [50] found differential expression of genes related with cellular metabolism, synthesis of protein and translation, cell cycle and DNA replication, and chitin and cuticular synthesis when comparing both morphologies. Such differential expression may even be responsible to trigger diapause in the winter morphology. Despite the genetic effect in the response to low temperatures, particularly about the overwinter, it was also suggested the importance of species behavior, suitable refuge sites and suitable food sources for population survival [51].

### Potential areas for invasion by *D*. *suzukii*

Both distribution models indicate areas with high environmental suitability for the occurrence of *D*. *suzukii* in the continents of Oceania and Africa, even though no records of the occurrence of the species exist. Models indicate that the environmental conditions in these areas are conducive to the establishment of the species in the event of a future invasion.

Our models suggest that the central region of Australia, due to the hot desert climate and the high rate of evaporation, is not suitable for the establishment of *D*. *suzukii*. However, areas with a subtropical climate with mild, wet summers are favorable for its establishment [36]. In addition, the North Island of New Zealand and Tasmania show high environmental suitability. Given their temperate maritime climates with cool, wet summers and rainfall distributed throughout the year, their provide favorable conditions for the establishment of *D*. *suzukii* in these locations. These regions may suffer economic damage from an invasion because they produce grapes, blueberries, blackberries, strawberries, gooseberries (*Ribes* sp.), figs and other soft-skinned fruits, which have been found to host *D*. *suzukii* in other countries [7, 52, 53, 54].

In Africa, our models indicate the areas with potential distribution are in western and eastern regions of South Africa and along a narrow range in the northern region of Africa. The area of highest environmental suitability occurs near the coast of Morocco, which has a temperate Mediterranean climate, with hot dry summers and wet and unstable winters [36]. In the southeastern region of Africa, our models indicate small areas of suitability in Mozambique and Madagascar, which have a tropical climate with high temperatures and rainfall [36].

## Conclusions

The models obtained in this study indicate potential areas that could be at risk of invasion by *D*. *suzukii* but that could also support national and international plans for pest management, thus avoiding the significant economic damage to fruit production for these continents, countries or states if establishment of this pest were to occur. There was a strong association between the areas of greatest environmental suitability as determined by the projections of the generated distribution models and areas with subtropical climates, because areas with mild temperatures and rainfall throughout the year are favorable to the establishment of *D*. *suzukii*.

## Supporting information

S1 File References used to compile the dataset. (DOCX)

S1 Table Results of the principal components analysis conducted to variables selection to insertion in the modeling. (XLSX)

S2 Table Correlation between the principal components obtained and the environmental variables values used to select the layers to modeling. (XLSX)

S3 Table Moran’s I values for the selected environmental variables and the respective standard deviations and p-values of the Monte Carlo tests for spatial autocorrelation. (XLSX)
