# Supplementary material for: Global potential distribution of Drosophila suzukii (Diptera, Drosophilidae)
Source: PLoS One. 2017 Mar 21;12(3):e0174318. doi: 10.1371/journal.pone.0174318 (PMC5360346; doi:10.1371/journal.pone.0174318)
Supplement: S1 File — (DOCX) [file pone.0174318.s001.docx]

**References used to compile the dataset**

Amin ud Din M, Khan SMN, Bakhsh A, Aleem R, Haque S, Salam A. Study of *Drosophila* association with certain plant species in Islamabad, Pakistan. *Drosoph* Inf Serv. 2005; 88: 15-16.

Amin ud Din M, Mazhar K, Haque S, Ahmed M. A preliminary report on *Drosophila* fauna of Islamabad (Capital, Pakistan). *Drosoph* Inf Serv. 2005; 88: 6-7.

Asquith A, Messing RH. Attraction of Hawaiian Ground litter invertebrates to protein hydrolysate bait. Environ Entomol.1992; 21: 1022-1028. doi:dx.doi.org/10.1093/ee/21.5.1022

Beardsley JW, Arakaki KT, Uchida, GK, Kumashiro BR. New records for Diptera in Hawaii. Bishop Mus Occ Pap. 1999; 58: 51-57.

Bitner-Mathé BC, Victorino J, Faria FS. *Drosophila suzukii* has been found in tropical Atlantic Rainforest in Southeastern Brazil. *Drosoph* Inf Serv. 2014; 97: 136-137.

Calabria G, Máca J, Bachli G, Serra L, Pascual M. First records of the potential pest species *Drosophila suzukii* (Diptera: Drosophilidae) in Europe. J appl Entomol. 2012; 134: 139-147. doi: 10.1111/j.1439-0418.2010.01583.x

Canals J, Balanya J, Mestres F. Drosophilid collection in the Font Groga site, Barcelona (Spain). *Drosoph* Inf Serv. 2013; 96: 185-186.

Carvajal JI. Genetic diversity of *Drosophila suzukii* in San Diego. *Drosoph* Inf Serv. 2010; 93: 67-68.

Castrezana S, Faircloth BC, Gowaty PA. *Drosophila* collection in Los Angeles, California. *Drosoph* Inf Serv. 2010; 93: 91-93.

Chabert S, Allemand R, Poyet M, Eslin P, Gibert P. Ability of European parasitoids (Hymenoptera) to control a new invasive Asiatic pest, *Drosophila suzukii*. Biol Control. 2012; 63: 40-47. doi: dx.doi.org/10.1016/j.biocontrol.2012.05.005

Chung YJ. Collection of Wild *Drosophila* on Quelpart Island, Korea. *Drosoph* Info Serv.1955; 29: 111.

Chung YJ. Drosophilid Survey of Ten Localities,South Korea.Korean J Zool.1958;1: 1-5. Available from: https://portal.koreascience.or.kr/article/articleresultdetail.jsp?no=HGDMB7_1958_v1n2_1

Chung YJ. Drosophilid Survey of two Islands in the South Sea of Korea. J. Korean Cult Res Inst. 1960; 2: 369-383. Available from: http://share.ewha.ac.kr/content/?p=000000006031

Chung YL, Lee KS. Further collection record of drosophilid flies from Korea. *Drosoph* Inf Serv. 1971; 46: 88.

Chung YL, Rho BJ. Drosophilid Survey at two Mountains in Kyung-pook province.J. Korean Cult Res Inst. 1960; 2: 385-393. Available from: http://share.ewha.ac.kr/content/?p=000000006032

Dalton DT, Walton VM, Shearer PW, Walsh DB, Caprile J, Isaacs R. Laboratory survival of *Drosophila suzukii* under simulated winter conditions of the Pacific Northwest and seasonal field trapping in five primary regions of small and stone fruit production in the United States. Pest Manag Sci. 2011; 67: 1368-1374. doi:10.1002/ps.2280

Deprá M, Poppe JL, Schmitz HJ, De Toni DC, Valente VLS. The first records of the invasive pest *Drosophila suzukii* in the South American continent. J Pest Sci. 2014; 87:379-383. doi:10.1007/s10340-014-0591-5.

Follett PA, Swedman A, Price DK. Postharvest irradiation treatment for quarantine control of *Drosophila suzukii* (Diptera: Drosophilidae) in fresh commodities. J Econ Entomol. 2014; 107: 964-969. doi:[dx.doi.org/10.1603/EC14006](http://dx.doi.org/10.1603/EC14006)

Gargani E, Tarchi F, Frosinini R, Mazza G, Simoni S. Notes on *Drosophila suzukii* Matsumura (Diptera, Drosophilidae): Field survey in Tuscany and laboratory evaluation of organic products. Redia J Zool. 2013; 96: 85-90.

Gonzalez G, Mary AL, Goni B. *Drosophila suzukii* (Matsumura) found in Uruguay. *Drosoph* Inf Serv. 2015; 98: 103-107.

Grassi A, Palmieri L, Giongo L. Nuovo fitofago per i piccolifrutti in Trentino. Terra Trentina.2009; 10: 19-23. Available from: http://hdl.handle.net/10449/19083

Hamby KA, Bolda MP, Sheehan ME, Zalom FG. Seasonal monitoring for *Drosophila suzukii* (Diptera: Drosophilidae) in California commercial raspberries. Environ Ent. 2014; 43: 1008-1018. doi:10.1603/EN13245

Hampton E, Koski C, Barsoian O, Faubert H, Cowles RS, Alm SR. Use of Early ripening cultivars to avoid infestation and mass trapping to manage *Drosophila suzukii* (Diptera: Drosophilidae) in *Vaccinium corymbosum* (Ericales: Ericaceae). J econ Entomol. 2014; 107: 1849-1857. doi:10.1603/EC14232

Hauser M, Gaimari S, Damus M. *Drosophila suzukii* new to North America. Fly Times. 2009; 43: 12-15.

Hirai Y, Goto SG, Yoshida T, Kimura MT. Faunal and Ecological Surveys on Drosophilid Flies in Iriomote-Jima, a Subtropical Island of Japan. Entomol Sci. 2000; 3: 273-284. Available from: http://ci.nii.ac.jp/naid/110003374667/

Hoddle M. Spotted wing *Drosophila* or Cherry Vinegar fly. Center for invasive species research. 2009. Available from: http://cisr.ucr.edu/spotted_wing_drosophila_cherry_vinegar_fly.html

Hu K, Carson HL. Interspecific copulation between far distant *Drosophila* species. *Drosoph* Inf Serv. 1987; 65: 75-76.

Iglesias LE, Price JF, Roubos CR, Renkema JM, Liburd OE, Price JF. Spotted wing Drosophila new in Florida Berry Culture. University of Florida IFAS Extension. 2009. Available from: http://edis.ifas.ufl.edu/pdffiles/IN/IN83900.pdf

Kaneshiro KY. *Drosophila* (*Sophophora*) *suzukii* (Matsumura). Proc Hawaii Entomol Soc. 1983; 24: 179.

Kang YS, Chung OK, Lee HY. Studies on the classification and the living conditions of Drosophilidae in Korea. Korean J Zool.1959; 2: 61-65. Available from: http://www.drosophila.jp/jdd/class/070301/07030137.pdf

Kang YS, Moon KW. Further notes of drosophilid fauna in the vicinity. *Drosoph* Inf Serv. 1969; 44: 62.

Kang YS, Moon KW. Drosophilid Fauna of Six Regions Near the Demilitarized Zone in Korea. Korean J Zool. 1968; 11: 65-68. Available from: http://www.koreascience.or.kr/article/ArticleFullRecord.jsp?cn=HGDMB7_1968_v11n2_65

Kawanishi M, Choo J, Nakamura K. Altitudinal and seasonal variations of the family Drosophilidae (Diptera) on the southern slope of Mt Fuji. Kontyû. 1975; 43: 106-116.

Kikkawa H, Peng FT. *Drosophila* Species of Japan and Adjacent Localities. Jpn J Zool. 1938; 7: 507-552.

Kim K-W. Drosophilidae of Tol-San Island Korea. *Drosoph* Inf Serv. 1953; 38:73.

Kim SS, Tripodi AD, Johnson DT, Szalanski AL. Molecular diagnostics of *Drosophila suzukii* (Diptera: Drosophilidae) using PCR-RFLP. J econ Entomol. 2014; 107: 1292-1294. doi:dx.doi.org/10.1603/EC13389

Kimura MT, Toda MJ. Drosophilid Fauna in Nakagawa Experiment Forest, Hokkaido University. Res Bull Coll exp For Hokkaido. 1976; 33: 449-456.

Kondo M, Kimura MT. Diversity of Drosophilid flies on Kume-Jima, a subtropical Island: comparison with diversty on Iriomote-Jima. Entomol Sci. 2008; 11: 7-15. doi:10.1111/j.1479-8298.2007.00250.x

Kwon OK, Toda MJ. A preliminary Study on the Ecological Structure of Note Drosophilid Community in the Quelpart Island, Korea, with a Supplementary Note on the Drosophilid Assemblage on tree Trunks. Nonmunjip Cheju Taehak. 1981; 13: 31-43.

Land M. Is there a new pest for Canada on the horizon? 2009. Available from: http://www.fruitandveggie.com

Landolt PJ, Adams T, Rogg H. Trapping spotted wing *Drosophila, Drosophila suzukii* (Matsumura) (Diptera: Drosophildae), with combinations of vinegar and wine, and acetic acid and ethanol. J appl Entomol. 2011; 136: 148-154. doi:10.1111/j.1439-0418.2011.01646.x

Leblanc L, O’Grady PM, Rubinoff D, Montgomery SL.New immigrant Drosophilidae in Hawaii, and a checklist of the Established immigrant species. Proc Hawaii Entomol Soc.2009; 41: 121-127.

Lee JC, Burrack HJ, Barrates LD, Beers EH, Dreves AJ, Hamby KA. et al. Evaluation of Monitoring Traps for *Drosophila suzukii* (Diptera: Drosophilidae) in North America. J econ Ent. 2012; 105: 1350-1357.

Lee TJ, Choo JK, Na DO, Kang KR. Studies on Ecological distribution of Drosophilidae in different localities of Korea. Kisul Kwahak Yon’guso. 1975; 2: 29-44.

Lee TJ, Kim NW. Drosophilidae collection from South Korea. *Drosoph* Inf Serv. 1991; 70: 127.

Lee TJ. A list of Drosophilid fauna in Korea. Chungang Univ Theses Coll.1966; 2: 7-20. Available from: http://www.drosophila.jp/jdd/class/070301/07030155.pdf

Lee TJ. Taxonomy and geographical distribution of Drosophilidae (Diptera) in Korea. Chungang Univ Theses Coll. 1964; 9: 425-459. Available from: <http://www.drosophila.jp/jdd/class/030706/03070608.pdf>

Lin FJ, Tseng HC, Lee WY. A catalogue of the family Drosophilidae in Taiwan (Diptera). Quarterly Journal of the Taiwan Museum. 1977; 30: 345-372. Available from: http://www.drosophila.jp/jdd/class/030707/03070757.pdf

Lin QC, Zhai YF, Zhang AS, Men XY, Zhang XY, Zalom FG, Zhou CG, Yu Y. Comparative Developmental Times and Laboratory Life Tables for *Drosophila suzukii* and *Drosophila melanogaster* (Diptera: Drosophilidae).Fla Entomol. 2014; 97: 1434-1442. doi: <http://dx.doi.org/10.1653/024.097.0418>

Maier CT. First detection and widespread distribution of the spotted wing *Drosophila*, *Drosophila suzukii* (Matsumura) (Diptera: Drosophilidae), in Connecticut in 2011. Proc Entomol Soc Wash. 2012; 114: 329-337. doi:dx.doi.org/10.4289/0013-8797.114.3.329

Makino S, Momma E, Takada H, Ishihara T. Species of *Drosophila* collected so far in Hokkaido, Japan (1952), by localities. *Drosoph* Inf Serv. 1952; 26: 109-110.

Mikasa K. A statistical analysis of seasonal fluctuations of population sizes in *Drosophila* populations near human habitation at Himeji City. (Abstract). Zool Sci Tokyo. 1990; 7: 1179.

Mortelmans J, Casteels h, Belien T. *Drosophila suzukii* (Diptera: Drosophilidae): A pest species new to Belgium. Belg J Zool. 2012; 142: 143-146.

Nicoladeli AT, Nunes HR, Ramirez MF, Cavalcante CJ, Carvalho-Pinto CJ, De Toni DC. First register of *Drosophila carcinophila* at South America, Brazil. *Drosoph* Inf Serv. 2014; 97:110-115.

O’Grady PM, Beardsley JW, Perreira WD. New records for introduced Drosophilidae (Diptera) in Hawaii. Bishop Mus Occ Pap. 2002; 68: 34-35.

Okada T. New distribution records of the drosophilids in the Oriental Region. (In Japonese). Makunagi. 1976; 8: 1-16. Available from: <http://www.drosophila.jp/jdd/class/030705/03070558.pdf>

Paik YK, Kim KW. Local Key to species of drosophilidae collected so far in South Korea. *Drosoph* Inf Serv. 1957; 31:153-154.

Paula MA, Lopes PHS, Tidon R. First Record of *Drosophila suzukii* in the Brazilian Savanna. *Drosoph* Inf Serv. 2014; 97: 113-115.

Roque F, Mencarini L, Tidon R. Revised list of drosophilid species recorded in the Brazilian Savanna. *Drosoph* Inf Serv. 2015; 98: 70-74.

Sidorenko VS. New and unrecorded species of Drosophilidae from Soviet Far East (Diptera, Brachycera). Spixiana. 1992; 15: 93-95.

Sidorenko VS. Tribe Drosophilini of the Asian part of the USSR (Diptera, Drosophilidae). Entomofauna. 1993; 14: 253-267. Available from: http://www.drosophila.jp/jdd/class/030711/03071115.pdf

Stewart TJ, Wang XG, Molinar A, Daane K. Factors limiting Peach as a potential Host for *Drosophila suzukii* (Diptera: Drosophilidae).J econ Ent. 2014; 107: 1771-1779. doi: http://dx.doi.org/10.1603/EC14197

Suss L, Costanzi M. Presence of *Drosophila suzukii* (Matsumura, 1931) (DipteraDrosophilidae) in Liguria (Italy). J Entomol Acar Res. 2010; 42: 185-188. doi:dx.doi.org/10.4081/jear.2010.185

Takada H, Lee TJ. A preliminary Survey of the Drosophilidae from Kongju and its Adjacent Localities, South Korea. Annot Zool Jpn**.** 1958; 31: 113-116.

Tiffany LA, Mc Robert SP. Population survey of *Drosophila* species in the Philadelphia area, Pennsylvania, U.S.A*. Drosoph* Inf Serv. 2013; 96: 179-180.

Tochen S, Dalton DT, Wiman N, Hann C, Shearer PW, Walton VM. Temperature-related development and population parameters for *Drosophila suzukii* (Diptera: Drosophilidae) on Cherry and Blueberry. Environ Entomol. 2014; 43: 501-510, doi:10.1603/EN13200

Toda MJ. Drosophilidae (Diptera) in Myanmar (Burma) VII. The *Drosophila melanogaster* species-group, excepting the *D. montium* species subgroup. Orient Insects. 1991; 25: 69-94. doi:10.1080/00305316.1991.10432216.

Toda MJ. Influence of Forest Felling upon Drosophilid Fauna at Several Localities in Hokkaido. Res Bull Coll Exp For Hokkaido. 1973; 30: 389-410. Available from: http://eprints.lib.hokudai.ac.jp/dspace/bitstream/2115/20928/1/30%282%29_P389-410.pdf

Vatte GS. Bilan de reseaux de piegeage *Drosophila suzukii*. Bull de Santedu Veg. 2011; 1: 1-5.

Vilela CR, Mori L. The invasive spotted-wing *Drosophila* (Diptera, Drosophilidae) has been found in the city of São Paulo (Brazil). Rev Bras Entomol. 2014; 58: 371-375. doi:dx.doi.org/10.1590/S0085-56262014000400004.

Wakahama K-I. Further note on the geographical distribution of the Drosophilidae in San-in Region. Bull Shimane Univ (Natural SCI.). 1962; 11: 41-46. Available from: http://www.lib.shimane-u.ac.jp/kiyo/b012/011/b004.pdf

Walsh D. Spotted wing *Drosophila* could pose threat for Washington fruit growers. Washington State University. 2009. Available from: http://ipm.wsu.edu/small/pdf/SWDrelease11-6-09.

Watada M, Morinaga K, Ochi O. Predominance of two colonizing species of *Drosophila* in Ehime Prefecture, Japan*. Drosoph* Inf Serv. 2000; 83: 93-100.

Watanabe TK, Kawanishi M. Geographical Distribution of *Drosophila simulans* in Japan. Zool Mag Tokyo. 1978; 87: 109-116.

Withers P,Allemand R.Les Drosophiles de la region Rhone-Alpes (Diptera, Drosophilidae). Bull Socentomol Fr. 2012; 117: 473-482.

Yamamoto AH. Niche differentiation of drosophilid in Oiso, Japan. Mem Konan Univ. 1992; 39: 287-299.

Yang Y, Zhang YP, Qian YH, Zeng QT. Phylogenetic relationships of *Drosophila melanogaster* species group deduced from spacer regions of histone gene H2A-H2B. Mol Phylogenet Evol. 2004; 30: 336-343.
